# Endocytosis, Cytotoxicity, and Translocation of Shiga Toxin-2 Are Stimulated by Infection of Human Intestinal (HCT-8) Monolayers With an Hypervirulent *E. coli* O157:H7 Lacking *stx*2 Gene

**DOI:** 10.3389/fcimb.2019.00396

**Published:** 2019-11-21

**Authors:** Nicolás Garimano, María Marta Amaral, Cristina Ibarra

**Affiliations:** Laboratorio de Fisiopatogenia, Departamento de Fisiología, Facultad de Medicina, Instituto de Fisiología y Biofísica Bernardo Houssay (IFIBIO Houssay-CONICET), Universidad de Buenos Aires, Buenos Aires, Argentina

**Keywords:** shiga toxin, HCT-8, STEC, O157, endocytosis, cytotoxicity, transcytosis, translocation

## Abstract

Shiga toxin-producing *Escherichia coli* (STEC) strains are responsible for multiple clinical syndromes, including hemolytic uremic syndrome (HUS). *E. coli* O157:H7 is the most prevalent serotype associated with HUS and produces a variety of virulence factors being Stx2 the responsible of the most HUS severe cases. After intestinal colonization by STEC, Stx2 is released into the intestinal lumen, translocated to the circulatory system and then binds to its receptor, globotriaosylceramide (Gb3), in target cells. Thus, Stx2 passage through the colonic epithelial barrier is a key step in order to produce disease, being its mechanisms still poorly understood. We have previously reported that STEC interaction with the human colonic mucosa enhanced Stx2 production. In the present work, we have demonstrated that infection with O157:H7Δstx2, a mutant unable to produce Stx2, enhanced either Stx2 cytotoxicity on an intestinal cell line (HCT-8), or translocation across HCT-8 monolayers. Moreover, we found that translocation was enhanced by both paracellular and transcellular pathways. Using specific endocytosis inhibitors, we have further demonstrated that the main mechanisms implicated on Stx2 endocytosis and translocation, either when O157:H7Δstx2 was present or not, were Gb3-dependent, but dynamin-independent. On the other hand, dynamin dependent endocytosis and macropinocytosis became more relevant only when O157:H7Δstx2 infection was present. Overall, this study highlights the effects of STEC infection on the intestinal epithelial cell host and the mechanisms underlying Stx2 endocytosis, cytotoxic activity and translocation, in the aim of finding new tools toward a therapeutic approach.

## Introduction

Shiga toxin-producing *Escherichia coli* (STEC) strains are responsible for multiple clinical syndromes including bloody diarrhea, hemorrhagic colitis, and hemolytic uremic syndrome (HUS) (Karmali et al., 1985). HUS is a systemic disease that can be fatal and is developed several days after STEC infection in up to 15% of children infected (Tarr et al., 2005). HUS is characterized by thrombotic microangiopathy, hemolytic anemia, thrombocytopenia, and acute renal failure (Gianantonio et al., 1968; Boyce et al., 2002). STEC are usually carried by cattle and bacterial ingestion frequently occurs via contaminated undercooked meat, unpasteurized dairy products, contaminated fruits, vegetables and water, and through animal to person or person to person contact (Ferens and Hovde, 2011). *E. coli* O157:H7 is the most prevalent serotype associated with HUS although multiple serotypes of STEC, including O157:NM strains and non-O157 serotypes such as O26:H11, O103:H2, O111:NM, O121:H19, and O145:NM have been associated with hemorrhagic colitis cases (Karmali et al., 2003). Some STEC strains commonly associated with serious disease possess a chromosomal pathogenicity island known as the locus of enterocyte effacement (LEE) (Nataro and Kaper, 1998), though LEE-negative strains which encode additional virulence, and colonization factors have also been associated with severe disease (Newton et al., 2009; Beutin and Martin, 2012; McWilliams and Torres, 2014). The genes encoded in the LEE are responsible for intimate adhesion of STEC to colonic epithelial cells (McWilliams and Torres, 2014), which is followed by injection of bacterial effector proteins into the host cell through a type III secretion system (T3SS) (Jerse et al., 1990). These effector proteins produce attaching and effacing (A/E) lesions on intestinal cells and interfere with host cells in many ways, inducing a profound rearrangement of cell cytoskeleton, and a loss of tight junction and membrane integrity (Knutton et al., 1989; Holmes et al., 2010; Ugalde-Silva et al., 2016). Additionally, STEC can produce either Stx1 and/or Stx2 prototypes, for which both have multiple subtypes (Melton-Celsa, 2014). Stx2 is widely recognized as the most important virulence factor of *E. coli* O157:H7 responsible for HUS (Palermo et al., 2009). This toxin is an AB5 toxin composed of an A subunit (Stx2A) and five B subunits (Stx2B). Stx2A possesses a N-glycosidase activity against 28S rRNA of 60S ribosomes in the cytosol. This activity results in an inhibition of protein synthesis in eukaryotic cells and activation of a proinflammatory signaling cascade known as the ribotoxic stress response, which is also involved in apoptosis induction (Smith et al., 2003).

On the other hand, Stx2B is arranged as pentamers of identical composition and has high affinity to the cell surface, glucosylceramide derived glycolipids, globotriaosylceramide (Gb3) and globotetraosylceramide (Gb4), though it has been found that only Gb3 may act as a functional receptor (Zumbrun et al., 2010). These glycolipids are generally located in cholesterol-rich cell membrane microdomains denominated lipid rafts (Hanashima et al., 2008; Legros et al., 2018) and are associated with toxin entry into target cells. Stx2 internalization has been shown to occur in two ways, one requiring specific binding of Stx2 to Gb3 receptor (Sandvig et al., 2002) and an unspecific macropinocytic pathway (Malyukova et al., 2009; Lukyanenko et al., 2011; In et al., 2013). Gb3 availability and microdomain location (Betz et al., 2011) and actin organization (Gaus et al., 2010) have been found to play a central role on Stx internalization. These factors are relevant on the mechanisms preceding endocytosis, as they impact clustering of Stx-Gb3 complexes on the cell membrane (Pezeshkian et al., 2017) and its capacity to ultimately form tubular invaginations prior to toxin internalization (Römer et al., 2007). The last step required for toxin internalization is vesicular scission, which has been found to occur via clathrin and dynamin (Lauvrak, 2004), dynamin only (Römer et al., 2007), and/or actin and cholesterol (Gaus et al., 2010) dependent mechanisms.

Several mechanisms for toxin translocation across intestinal epithelium have been proposed. Non-Gb3 associated translocation has been found to occur via the paracellular pathway, stimulated by neutrophil transmigration and actin rearrangements during STEC infection (Hurley et al., 2001), but also via the transcellular pathway, implicating an unspecific macropinocytic mechanism that does not involve the Gb3 receptor (Lukyanenko et al., 2011). There is also evidence that Stx2 transcellular transcytosis may be due to Gb3-linked Stx2 endocytosis, as it was described that a percentage of apical recycling endosomes containing Stx2 are released on the basolateral side (Müller et al., 2017), although Gb3 presence on colonic epithelium is still under debate (Schüller et al., 2004; Zumbrun et al., 2010). Previous studies have described that these routes can be selectively and efficiently inhibited (Macia et al., 2006; Huang et al., 2010; Koivusalo et al., 2010; Dutta and Donaldson, 2012; Shayman, 2013; Kaissarian et al., 2018), even though their particular relevance to Stx2 endocytosis and translocation is still unclear.

*In vitro* studies using human intestinal epithelial cell lines have demonstrated that epithelial cell infection with STEC may lead to enhanced Stx2 translocation depending on the strain virulence profile (Tran et al., 2018). In turn, intestinal epithelial cell-derived molecules could also modify STEC virulence by increasing adhesion and upregulating virulent genetic profiles (Bansal et al., 2013). In our previous work, we investigated the effect of *E. coli* O157:H7 expressing Stx2 on human colonic mucosa and showed that bacterial interaction with colonic epithelium enhanced Stx2 production that, in turn, caused a marked inhibition of water absorption concomitant with histological damages on the surface epithelium (Albanese et al., 2015). In this study, we analyze the effects of *E. coli* O157:H7Δstx2 infection on Stx2 cytotoxic effects, endocytosis and translocation across polarized HCT-8 cells, which express Gb3 and Gb4 receptors (Kouzel et al., 2017). To this aim, we used specific endocytosis inhibitors, in order to demonstrate which pathways are stimulated upon infection, providing an *in vitro* basis to further develop therapeutic targets.

## Materials and Methods

### Materials

Purified Stx2a was provided from Phoenix Laboratory (Tufts Medical Center, Boston, MA, USA). Eliglustat (Cerdelga, Sanofis-Genzyme) used as an inhibitor of glucosylceramide synthase (Shayman, 2013) was purchased from MedKoo Biosci, Morrisville, USA. Dynasore, a specific dynamin-mediated endocytic inhibitor (Macia et al., 2006), Methyl-β-cyclodextrin (MβCD), a membrane cholesterol extractor (Zidovetzki and Levitan, 2007), and Amiloride hydrochloride, a macropinocytosis inhibitor (Koivusalo et al., 2010) were purchased from Sigma Aldrich, St. Louis, MO, USA. A fluorescein isothiocyanate (FITC)-labeled Dextran (average molecular weight of 70 kDa, Sigma Aldrich, catalog # 46945) was used as a marker of paracellular permeability (Chattopadhyay et al., 2017). A mouse monoclonal antibody against the A-subunit of Stx2 (Mab 2E11) was provided by Roxane Piazza (Butantan Institute, São Paulo, SP, Brazil) (Rocha et al., 2012) and an Alexa 647-conjugated anti-mouse secondary antibody (AbCam, catalog #ab150115) were used for flow cytometry.

### Bacterial Strains and Growth Conditions

*E. coli* O157:H7 strain 125/99 wild type (O157:H7) isolated from a patient with HUS has been previously described (Rivas et al., 2006). A mutant lacking *stx2* gene from the parenteral *E. coli* O157:H7 strain 125/99wt (O157:H7Δstx2) was previously obtained (Albanese et al., 2015). O157:H7Δstx2 was grown in Luria Broth medium for 18 h at 37°C with shaking at 150 rpm and then diluted 1/10 in DMEM/F12 medium (Sigma Aldrich, USA) with the addition of HEPES 10 mM and grown to exponential phase (OD_600_ = 0.3–0.4) at 37°C with shaking at 150 rpm. O157:H7Δstx2 density corresponded to ~4 × 10^9^ colony-forming units per ml (CFU/ml). O157:H7Δstx2 supernatant (SN O157:H7) was collected after centrifugation at 10,000 g for 5 min, followed by filtration through a 0.22-μm filter (Millipore, Billerica, MA, USA).

### Cell Cultures

The human intestinal cell line HCT-8 (ATCC CCL-244, Manassas, VA, USA) was maintained in RPMI-1640 medium (ATCC) and the monkey kidney cell line Vero (ATCC CCL-81) was grown in DMEM/F12 (Sigma Aldrich, St. Louis, MO USA). Both media were supplemented with 10% fetal bovine serum (FBS, Internegocios S.A., Buenos Aires, Argentina), 100 U/ml penicillin and 100 μg/ml streptomycin. Additionally, 1 mM L-glutamine, 10 mM sodium pyruvate, 10 mM HEPES, 10 mM glucose were also supplemented in HCT-8 cultures. Cells were grown at 37°C in a humidified 5% CO_2_ incubator. Cells were subcultured after 80% confluence was reached (7–10 days) in antibiotic-free medium. During experiments, HCT-8 cells were maintained in growth-arrested conditions (medium without FBS).

### Cytotoxicity and Neutralization Assay on HCT-8 Cells

Purified Stx2 and O157:H7Δstx2 induced cytotoxicity were assayed on HCT-8 cells cultured on fixed support. Briefly, HCT-8 cells monolayers grown on 96-well plates were treated for 4 h under growth-arrested conditions (Culture media without FBS) and antibiotic free conditions with 100 ng/ml Stx2 and/or 4 × 10^8^ CFU/ml O157:H7Δstx2. In selected experiments, cells were preincubated with Eliglustat (200 nM, 48 h Kaissarian et al., 2018), Dynasore (80 μM, 30 min), MβCD (4 mM, 30 min), or Amiloride (1 mM, 30 min) and washed twice with PBS before treatment. At the end of the incubation time, plates were washed twice with PBS (145 mM NaCl, 10 mM NaH_2_PO_4_, pH 7.2) and subsequently incubated in growth-arrested media for 72 h. Then, cell viability was assayed by neutral red uptake (Repetto et al., 2008).

Cytotoxic activity neutralization was calculated using the following equation.

(1) $$\begin{array}{r} Cytotoxic\;Activity\;Neutralization=100*\frac{I-T}{100-T} \end{array}$$

In this Equation, I and T are % cell viability of HCT-8 cells pre-treated or not with endocytosis inhibitors respectively.

### Neutral Red Uptake Assay

Neutral red uptake assay was performed as previously described, with minor modifications (Repetto et al., 2008). After treatment, cells were washed twice with PBS (145 mM NaCl, 10 mM NaH_2_PO_4_, pH 7.2) and incubated for 2 h with freshly diluted neutral red in PBS to a final concentration of 50 μg/ml. Cells were then washed with 1% CaCl_2_ and 4% formaldehyde twice and were then solubilized in 1% acetic acid and 50% ethanol. Absorbance at 546 nm was read in an automated plate spectrophotometer. Results were expressed as percent viability, with 100% represented by cells incubated under identical conditions but without treatment. The 50% cytotoxic dose (CD_50_) corresponded to the dilution required to kill 50% of the cells.

### Cell Monolayer Culture

HCT-8 were seeded on Milicell culture inserts (PIHP01250, Millipore, Billerica, MA, USA) of 12 mm diameter and 0.4 um pore size (filter area: 1.13 cm) placed on a 24-well plate and grown for about 7–10 days as previously described until a continuous monolayer was achieved. The development of monolayers was monitored with the electrical resistance (TEER) measured with a Millicell-ERS electric resistance system (Millipore, Billerica, MA, USA) until TEER values were stable for 2 consecutive days and higher than 1,200 Ω.cm^2^, which is consistent with cell polarization (Hurley et al., 1999).

### Translocation Assays

Briefly, after HCT-8 cell monolayer formation, complete medium was removed from upper (apical) and lower (basolateral) chambers and replaced with growth-arrested and antibiotic free medium. Apical side of the HCT-8 cells were exposed to Stx2 (100 ng/ml) in the absence or presence of O157:H7Δstx2 (4 × 10^8^ CFU/ml), its filtered supernatant SN-O157:H7Δstx2 (1:10 diluted) or EDTA (10 mM) as a tight junction disruptor for 4 h at 37°C in 5% CO_2_ atmosphere. Following the incubation period, the media from the lower chamber was filter-sterilized to determine Stx2 concentration by Vero cell cytotoxic assays. In selected experiments, cells were preincubated with Eliglustat (200 nM, 48 h), Dynasore (80 μM, 30 min), MβCD (4 mM, 30 min), or Amiloride (1 mM, 30 min) and washed twice with PBS before treatments were applied.

### Stx2 Quantification by Vero Cell Cytotoxicity Assay

Vero cells grown in 96-well culture plate until confluence were treated in serum-free medium for 24 h with different concentrations of purified Stx2 or different dilutions of experimental samples. At the end of the incubation, cell viability was analyzed by neutral red uptake. Stx2 concentrations were calculated from Stx2 standard curves. When the cytotoxic effect elicited by filter-sterilized SN from O157:H7 grown to exponential phase was compared to a purified Stx2 curve used as standard, the average cytotoxicity of the filtered SN was equivalent to 100 ± 6 ng/ml Stx2 (data not shown).

### Paracellular Permeability

Paracellular permeability in HCT-8 monolayers was determined by measuring FITC-Dextran passage from the apical- to basolateral side of monolayers, taking into account that Dextran (MW 70 kDa) cannot penetrate the cellular membrane under physiological conditions being its molecular weight similar to Stx2 (Hashida et al., 1986; Balda et al., 1996; Matter and Balda, 2003). FITC-Dextran was measured according to methods described previously with some modifications (Balda et al., 1996). FITC-Dextran (1 mg/ml) was added on the upper (apical) chamber at the beginning of each experiment. Following the incubation period, 100 μl of media from the upper (apical) and lower (basolateral) chamber collected separately were placed in a 96-well plate and the concentration of FITC-Dextran was measured on a fluorescence multiplate reader (FLUOstar Omega, excitation, 486 nm; emission, 520 nm). Relative fluorescence was then calculated as a ratio between lower (basolateral) chamber fluorescence and total fluorescence. Sample readings were performed in triplicate.

### Adhesion of O157:H7Δstx2 to HCT-8 Cells

HCT-8 cells were grown on 24-wells plates until a confluent monolayer was achieved. Monolayers were pretreated with Eliglustat (200 nM, 48 h), Dynasore (80 μM, 30 min), MβCD (4 mM, 30 min), or Amiloride (1 mM, 30 min), washed twice with PBS and then treated for 4 h with 4 × 10^8^ CFU/ml O157:H7Δstx2 in the presence of 100 ng/ml Stx2. To count CFU number, cells were washed 5 times with PBS to remove non-attached bacteria and lysed using 0.2% Triton-PBS solution for 30 min. Serial dilutions of these suspensions were spread on LB-agar coated Petri dishes and incubated at 37°C for 24 h for optical counting.

### Stx2 Flow Cytometry Detection

The presence of intracellular and extracellular Stx2 in HCT-8 cells pre-incubated with endocytic inhibitors was evaluated in HCT-8 cells by flow cytometry. For that purpose, cells were fixed after treatment in 0.5% paraformaldehyde, and for intracellular staining, permeabilized with saponin 0.1% in phosphate-buffered saline (PBS). Then, cells were incubated in PBS 0.5% FBS with Mab 2E11 for 2 h, followed by a 1 h incubation with an Alexa 647-conjugated anti-mouse secondary antibody for 1 h. Cells were subsequently washed and resuspended in PBS. The staining was analyzed by flow cytometry on PARTEC PAS III using Cyflogic software 1.2.1. Results are expressed as percentage of Stx2 positive events and median intensity of fluorescence (MIF) in cells treated compared to cells not pre-treated with inhibitors.

### Statistical Analysis

Cytotoxicity curves were fitted using linear or logarithmic regression. Statistical significance for all assays was assessed using one-way ANOVA with Tukey's or Bonferroni's multiple comparison test or Student's *t*-test. Analysis was performed using Graphpad Prism v6.01 software. Statistical significance was set at ^*^p < 0.05.

## Results

### Effects of O157:H7Δstx2 Infection on Stx2 Cytotoxicity

**Figure 1:**
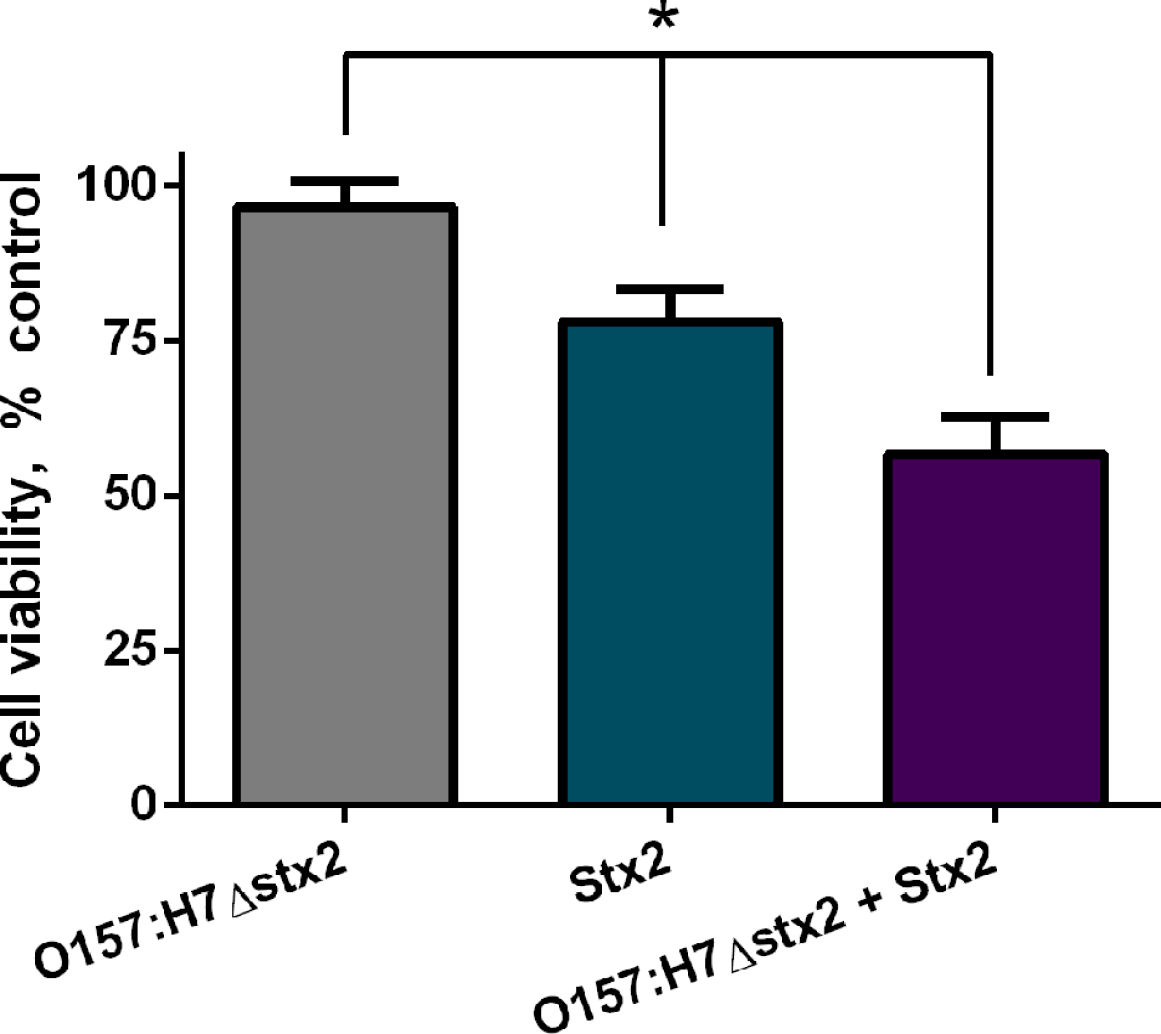
Stx2 and O157:H7Δstx2 cytotoxicity on HCT-8 cells. HCT-8 cells were incubated with either 100 ng/ml Stx2 alone or in the presence of 4 x 10^8^ CFU/ml O157:H7Δstx2 (O157:H7Δstx2 + Stx2) for 4 h and compared with 4 × 10^8^ O157:H7Δstx2 alone. HCT-8 viability was measured by neutral red uptake after 72 h. Data is shown as means ± SEM from six independent experiments performed in triplicate. Stx2 vs. O157:H7Δstx2 or Stx2 vs. O157:H7Δstx2 + Stx2. ^*^p < 0.05.

To examine if Stx2 cytotoxicity could be modulated by O157:H7Δstx2 infection, cell viability was measured on HCT-8 cells incubated with either 100 ng/ml Stx2 alone or in presence of 4 × 10^8^ CFU/ml O157:H7Δstx2 (O157:H7Δstx2+Stx2) and compared with HCT-8 cells incubated with O157:H7Δstx2 alone. As shown in Figure 1, the cytotoxic effect caused by O157:H7Δstx2 + Stx2 was significantly greater compared to Stx2 alone, while O157:H7Δstx2 did not show any cytotoxic effect. These results suggest that an increase in Stx2 cytotoxicity is elicited by O157:H7Δstx2 infection.

### Effect of Endocytic-Pathway Inhibitors on Stx2 Uptake and Cytotoxic Effects

**Figure 2:**
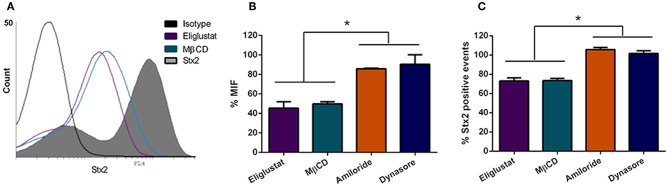
Stx2 uptake by HCT-8 cells. **(A)** Cells pre-treated or not with endocytosis inhibitors were exposed to 100 ng/ml Stx2 and then analyzed by flow cytometry. Histograms represent the log fluorescence of Stx2 for each treatment. A Representative experiment is shown. Amiloride and Dynasore curves are not shown due to superimposition with Stx2 curve. **(B)** Bar graph representing the Median Intensity of Fluorescence (% MIF) for each inhibitor treatment relative to cells only treated with Stx2. **(C)** Stx2 positive events (%) for each inhibitor treatment relative to cells only treated with Stx2. Bars represent the mean ± SEM of three independent experiments. ^*^p < 0.05.

The finding that O157:H7Δstx2 infection significantly increased Stx2 cytotoxicity in HCT-8 cells led the studies to determine which pathways are involved in Stx2 uptake and cytotoxicity and, in turn, which ones were stimulated by O157:H7Δstx2 infection. HCT-8 cells were pre-treated with Eliglustat (200 nM, 48 h), an inhibitor of glucosylceramide (GL1) synthase (Shayman, 2013), MβCD (4 mM, 30 min), a membrane cholesterol extractor (Zidovetzki and Levitan, 2007), Dynasore (80 μM, 30 min), a specific dynamin-mediated endocytic inhibitor (Macia et al., 2006) or Amiloride (1 mM, 30 min), a specific dynamin-mediated endocytic inhibitor (Macia et al., 2006). Cells were then incubated with 100 ng/ml Stx2 during 4 h. Finally, toxin uptake was measured by flow cytometry. As it can be seen on Figure 2, both % MIF and % Stx2 positive events were significantly lower when cells were pre-incubated with Eliglustat or MβCD, compared to Dynasore or Amiloride, indicating that Stx2 uptake may be sensitive to glucosylceramide synthase inhibition (presumably due to Gb3 synthesis inhibition) and/or cholesterol dependent but not significantly dynamin or macropinocytosis dependent.

**Figure 3:**
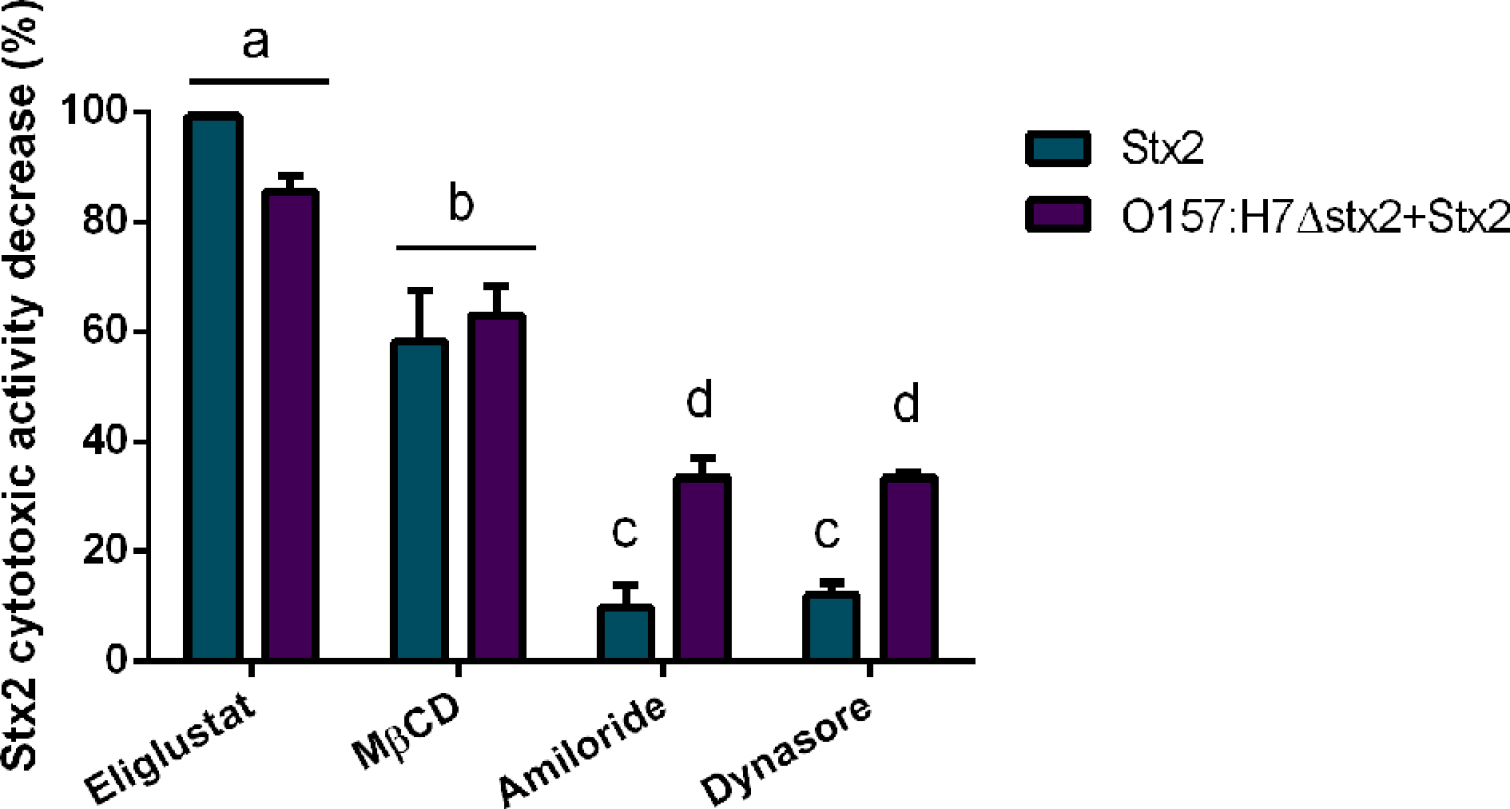
Effect of endocytosis inhibitors on Stx2 cytotoxicity in HCT-8 cells. Cells were pre-incubated with Eliglustat (200 nM, 48 h), MβCD (4 mM, 30 min), Dynasore (80 μM, 30 min), or Amiloride (1 mM, 30 min) and washed twice with PBS before treatment for 4 h of 100 ng/ml Stx2 with or without O157:H7Δstx2. Data is shown as means ± SEM from six independent experiments performed in triplicate. Significant differences were found between the groups, labeled a, b, c, and d.

In agreement with the reduced level of Stx2 uptake observed following inhibitor treatments, Stx2 cytotoxicity was also decreased by the inhibitors Eliglustat and MβCD (Figure 3), with Eliglustat having a more pronounced effect.

Notably, the neutralizing effect on cytotoxicity by Eliglustat was maximal when O157:H7Δstx2 were not present and appeared lower when O157:H7Δstx2 were present, though no statistically significant differences were found. On the other hand, Amiloride and Dynasore showed a significantly lower protective capability than Eliglustat and MβCD, but both showed a significantly higher neutralizing capability when cells were treated with O157:H7Δstx2+Stx2 compared to Stx2 alone (Figure 3). None of these inhibitors showed cytotoxic activity *per se* when they were tested alone (data not shown). This data is consistent with a necessary interaction between Stx2 and the Gb3 receptor, presented in the apical surface of HCT-8 cells, to cause cytotoxicity, although these effects appear to be less dependent on the lipid environment compared to the net Stx2 uptake measured by flow cytometry. However, dynamin-dependent and Gb3-independent macropinocytic endocytosis pathways became relevant only when O157:H7Δstx2 were present, suggesting that these mechanisms are sensitive to O157:H7Δstx2 infection.

None of the inhibitors used for pre-incubation showed a significant effect on O157:H7Δstx2 adhesion to HCT-8 cells (Figure S1).

### Effect of O157:H7 Infection on Stx2 Translocation

**Figure 4:**
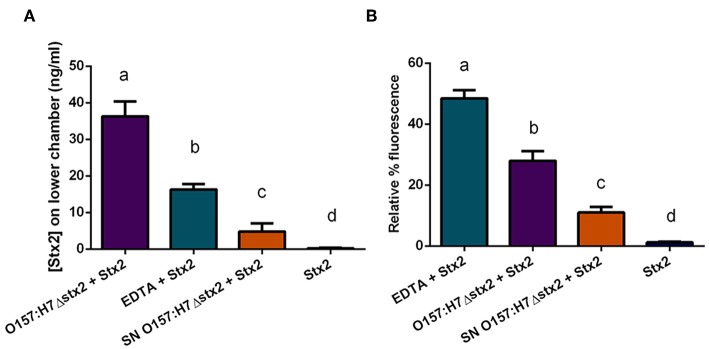
Stx2 translocation across HCT-8 monolayers. **(A)** Stx2 concentration in the lower (basolateral) chamber was determined by Vero cell cytotoxicity assays. **(B)** Relative FITC-Dextran passage (%) to the lower (basolateral) chamber was measured. Significant differences were found between the groups, labeled a, b, c, and d.

To determine whether Stx2 translocation across the intestinal barrier was affected by the presence of O157:H7Δstx2, HCT-8 monolayers were incubated with 100 ng/ml of Stx2 alone or in the presence of: 4 x 10^8^ CFU/ml O157:H7Δstx2, 10 mM EDTA, or O157:H7Δstx2 supernatant from O157:H7Δstx2 (SN O157:H7Δstx2), added on the upper (apical) chamber. After 4 h of incubation, Stx2 passage was quantified on the lower (basolateral) chamber by Vero cell cytotoxicity assay (Figure 4A).

Since Stx has been shown to be able to cross polarized epithelial cells through both transcellular and paracellular pathways (Hurley et al., 2001; Malyukova et al., 2009; Lukyanenko et al., 2011; Tran et al., 2018), FITC-Dextran passage from apical to basolateral side was simultaneously measured (Figure 4B). Maximum Stx2 translocation across the HCT-8 monolayers was found in the presence of O157:H7Δstx2 compared to the other experimental conditions (Figure 4A). Instead, maximum FITC-Dextran passage was found in the presence of EDTA, a potent tight junction disruptor agent, followed by O157:H7Δstx2 treatment (Figure 4B). Taken together, these results have demonstrated that O157:H7Δstx2 stimulates not only paracellular, but also transcellular Stx2 translocation.

On the other hand, monolayers exposed to SN O157:H7Δstx2 also resulted in a significant increase of Stx2 and FITC-Dextran passage (Figures 4A,B, respectively) indicating that O157:H7Δstx2 SN affected at least the paracellular permeability but to a lesser extent than that observed with O157:H7Δstx2 (Figure 4Bb vs. c), compared to treatment with Stx2 alone (Figure 4Bd).

**Figure 5:**
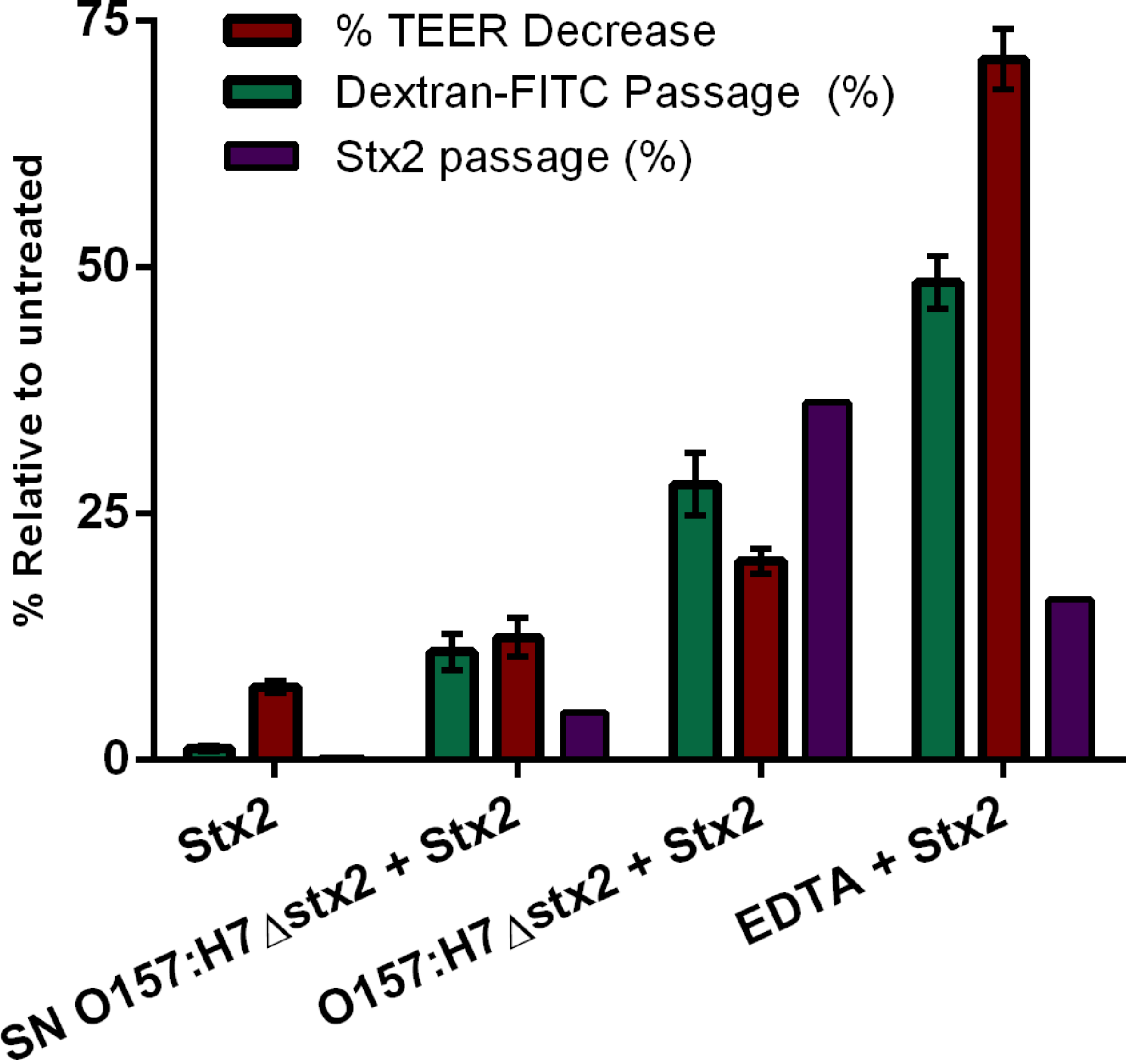
Stx2 translocation was not entirely correlated to tight junction barrier disruption. The correlation between Stx2 translocation and the disruptive effects on tight junctional barrier measured as TEER and FITC-Dextran passage was analyzed in HCT-8 cell monolayers after 4 h incubation with O157:H7Δstx2, SN O157:H7Δstx2 and EDTA. Stx2 concentration was determined in the lower (basolateral) chamber by Vero cell cytotoxicity assays and it was relativized to initial Stx2 concentration (100 ng/ml). Relative FITC-Dextran passage was calculated as a ratio between lower (basolateral) chamber fluorescence and total fluorescence relative to cell free control. TEER was measured at the end of treatments and expressed as a percentage of the corresponding TEER immediately before the start of treatments.

To confirm the effects of O157:H7Δstx2 and its filtered SN on epithelial barrier function leading to Stx2 paracellular translocation, we analyzed the integrity of the tight junctional barrier by TEER measurements before and after each treatment (Figure 5). The integrity of tight junctions clearly declined in monolayers treated with O157:H7Δstx2 and SN O157:H7Δstx2 in the presence of Stx2, proportionally to the increase of FITC-Dextran passage. As it was expected, minimum TEER value was observed after incubation with 10 mM EDTA. However, the Stx2 passage was higher in the presence of O157:H7Δstx2 than with EDTA, supporting the hypothesis that O157:H7Δstx2 stimulates Stx2 translocation across transcellular and paracellular pathways.

### Effects of Endocytosis Inhibitors on Stx2 Passage Across the Transcellular Pathway

**Figure 6:**
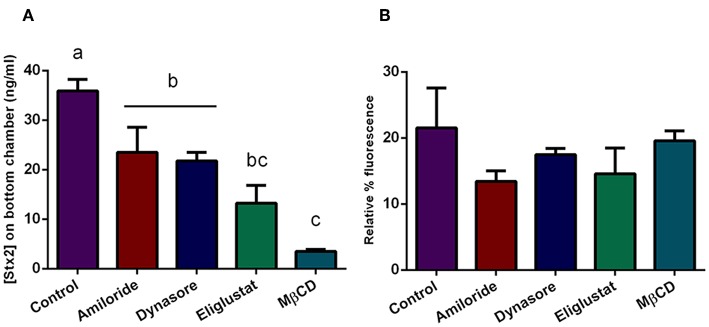
Stx2 translocation in presence of endocytosis inhibitors. Cells were pre-incubated with Eliglustat (200 nM, 48 h), MBCD (4 mM, 30 min), Dynasore (80 μM, 30 min), or Amiloride (1 mM, 30 min), and washed twice with PBS followed by treatment with O157:H7Δstx2 + 100 ng/ml Stx2 for 4 h. Control cells were not pre-treated with inhibitors. **(A)** Estimated Stx2 concentration in the lower (basolateral) chamber. **(B)** Relative FITC-Dextran passage (%) to the lower (basolateral) chamber. Significant differences were found between groups with different letters, labeled a, b, and c.

To assess which endocytic mechanisms previously described are involved in Stx2 transcytosis in presence of O157:H7Δstx2, HCT-8 cells monolayers were pre-incubated with Eliglustat, Dynasore, MβCD, or Amiloride as previously described, followed by incubation with O157:H7Δstx2+Stx2. Stx2 translocation, measured as indicated above, was significantly inhibited by all treatments, with a maximum inhibition observed after MβCD treatment, followed by Eliglustat, and lastly, Dynasore and Amiloride (Figure 6A). In addition, none of these inhibitors significantly disrupted the epithelial barrier function measured as FITC-Dextran translocation (Figure 6B). These results suggest that cholesterol disruption was more efficient than glucosylceramide synthesis inhibition at decreasing endocytosis-dependent translocation, suggesting a wider implication of cholesterol in the transcytosis process. In addition, Stx2 translocation via dynamin and macropinocytic pathways was also significantly decreased, showing that both mechanisms also may play a role on Stx2 transcytosis during O157:H7Δstx2 infection.

## Discussion

We have previously reported that Stx2-mediated physiological and morphological alterations to human colonic mucosa was enhanced by exposure to O157:H7 bacteria (Albanese et al., 2015). In the present study, we demonstrate that infection of human colonic epithelial (HCT-8) monolayers by O157:H7Δstx2 also impacts Stx2 endocytosis, cytotoxic action, and translocation across intestinal epithelial monolayers.

In this report, HCT-8 cells infected with O157:H7Δstx2 supplemented with Stx2 exhibited a significantly higher cytotoxic activity compared to the same concentration of Stx2 alone, indicating that O157:H7Δstx2 infection led to host cell modifications that enhanced Stx2 cytotoxicity (Figure 1). Regarding Stx2 effects on uninfected cells, we have seen that inhibition of glucosylceramide synthesis by Eliglustat led to a maximum decrease in Stx2 entry into cells and cytotoxic effects on HCT-8 cells (Figures 2, 3). This inhibitor has been used at optimal concentration and incubation time according to previous works with endothelial cells (Kaissarian et al., 2018) and to previous experience with another glucosylceramide synthase inhibitor (Miglustat, Acetilon Pharm) (Girard et al., 2015) that also reduces Gb3 synthesis. It is worth noting that Eliglustat inhibits the synthesis of multiple glucosylceramides besides Gb3, some of which have been implied in membrane traffic and endocytic pathway modulation (Sillence et al., 2002). In this direction, it is possible that the absence of these glucosylceramides may account for some of the effects observed, but we speculate that their relative relevance on Stx2 uptake and cytotoxic activity should be minor compared to Gb3 absence. Our results indicate that, although Eliglustat prevented Stx2 cytotoxic effects (Figure 3), it did not completely inhibit net Stx2 internalization (Figure 2). We propose that this observed Stx2 uptake may be due to Gb3-independent endocytic mechanisms (Chan and Ng, 2016), although it may not have been enough to exert Stx2 cytotoxic effects. Similarly, removal of cholesterol by using MβCD reduced Stx2 uptake and cytotoxicity, although it appeared to do so to a lesser extent than that observed with Eliglustat. Cholesterol is known to be a major component of the microenvironment in which Gb3 is embedded, and it has previously been implicated on Gb3 avidity for Stx2 (Gallegos et al., 2012) and Stx2-induced tubule scission (Gaus et al., 2010). It was also suggested that cholesterol may play a role on macropinocytosis and membrane ruffling (Grimmer et al., 2002). Although significant differences were not found, Eliglustat appeared to be less effective on decreasing Stx2 cytotoxicity when O157:H7Δstx2 was present, compared to the bacterial-free condition (Figure 3). Even though the methods used may have not been sensitive enough to provide significant differences in this scenario, we speculate that this result is relevant as it may account for the significant stimulation observed of alternative mechanisms, such as macropinocytosis and dynamin-dependent pathways, upon O157:H7Δstx2 infection. On the other hand, dynamin's role on Stx2 induced tubule scission after Gb3 binding is highly controversial, as a putative spontaneous, actin-dependent mechanism has also been described (Gaus et al., 2010). In this study, we found that dynamin-dependent Stx2 endocytosis had no relevance on Stx2 uptake (Figure 2) or cytotoxic effects when O157:H7Δstx2 was absent but did significantly contribute to Stx2 cytotoxicity upon O157:H7Δstx2 infection (Figure 3). We speculate that these differences may be explained as it was observed that O157:H7Δstx2 infection could exert a positive action on dynamin recruitment around vesicles, which is required for actin pedestal formation (Unsworth et al., 2007). Alternatively, we hypothesize that actin rearrangement induced by O157:H7Δstx2 may have affected actin-dependent vesicle scission mechanisms (Gaus et al., 2010), which may in turn increase dynamin dependent scission, although the exact underlying causes remain a subject for future studies. Macropinocytosis involvement in Stx2 internalization during STEC infection and the molecular mechanisms implicated has been previously described (Malyukova et al., 2009; Lukyanenko et al., 2011; In et al., 2013). In this study, we found that macropinocytosis significantly contributed to Stx2 internalization only when O157:H7Δstx2 were present, indicating that bacterial infection exerts a positive effect on cell's macropinocytosis regulation (Figure 3).

As O157:H7 is generally believed to be non-invasive, Stx2 must translocate across the epithelial barrier of the intestine in order to reach target organs and cause HUS (Boyce et al., 2002). In this study, we demonstrate that Stx2 passage across polarized HCT-8 monolayers is enhanced by O157:H7Δstx2 infection. This increase is not exclusively due to soluble metabolites, as SN O157:H7Δstx2 exerted a much lesser, although statistically significant, effect on transcytosis (Figure 4A). Previous studies have shown that Stx2 may translocate the intestinal barrier by a paracellular in addition to a transcellular/endocytic pathway (Hurley et al., 2001; Müller et al., 2017). We used EDTA, a chelating agent extensively described as a tight junction disruptor (Ward et al., 2000), to estimate the maximum passage allowed by the paracellular route. We confirmed by TEER and FITC-Dextran measurements that paracellular passage and permeability were maximum when EDTA was added, followed by O157:H7Δstx2 treatment (Figures 4B, 5). However, Stx2 translocation in presence of O157:H7Δstx2 was significantly higher than with EDTA treatment (Figure 5), suggesting that both paracellular and transcellular translocation routes can be upregulated by O157:H7Δstx2. Again, SN O157:H7Δstx2 was able to exert a significant effect over tight junction integrity compared to Stx2 alone, but much less than that exerted by O157:H7Δstx2 infection. Previous reports showed that Stx2 translocation across a polarized colonic epithelial T84 cell line is enhanced by O157:H7 infection and may only occur via a transcellular pathway because TEER remains constant during infection with several STEC O157:H7 strains (Tran et al., 2014, 2018). In the same fashion, we found that the transcellular pathway is stimulated by O157:H7Δstx2 infection, but we also observed that the paracellular pathway, measured as TEER and FITC-Dextran permeability, was significantly affected after infection with O157:H7Δstx2 (Figure 5). The apparent discrepancy could be due to differences in STEC strains, as the one used in this study belongs to the hypervirulent clade 8, which was found to overexpress several virulence proteins (Amigo et al., 2015, 2016). Differences in intestinal cell lines used may also explain these discrepancies, as they show differences in key characteristics such as monolayers TEER values (Hurley et al., 2016), which we speculate may account for differences in sensitivity of tight junction to O157:H7 infection.

Overall, our data suggests that O157:H7Δstx2 infection greatly stimulated Stx2 passage through both paracellular and transcellular pathways, and that this stimulation is mainly due to O157:H7Δstx2 co-incubation with epithelial cells, and to a lesser extent, to soluble metabolites released by O157:H7Δstx2 to the culture supernatant.

We also tested if the same endocytic pathways involved in cytotoxicity could account for the increased transcytosis. Surprisingly, we found that cholesterol depletion by MβCD appears to be more effective decreasing Stx2 transcytosis than glucosylceramide synthesis inhibition by Eliglustat (Figure 6), though the opposite was observed when cytotoxic effects were analyzed (Figure 3). Being the exact mechanisms that determine vesicle trafficking and its destiny still unknown, we can hypothesize that membrane cholesterol may be more strongly implied on the basolateral trafficking leading to translocation, rather than on the retrograde transport leading to cytotoxicity. On the other hand, Amiloride and Dynasore led to a moderate transcytosis decrease (Figure 6) in the same fashion as that observed in cytotoxic neutralization (Figure 3), suggesting that O157:H7Δstx2 could also stimulate macropinocytosis and dynamin-dependent translocation. As these endocytosis inhibitors showed similar effects on both cytotoxicity and transcytosis, we can hypothesize that, to a degree, the mechanisms leading to both outcomes may have the same endocytic origin (Malyukova et al., 2009; Müller et al., 2017).

To summarize, our results showed that *E. coli* O157:H7Δstx2 infection increases Stx2 cytotoxic effect by stimulation of several endocytic pathways. Furthermore, we demonstrated that O157:H7Δstx2 infection enhances Stx2 translocation across HCT-8 monolayers in both paracellular and transcellular pathways. We showed that dynamin-independent and Gb3-dependent mechanisms are mostly implicated in Stx2 endocytosis and translocation, though dynamin dependent endocytosis and macropinocytosis became more relevant when O157:H7Δstx2 infection was present.

This study provides insight on how O157:H7 infection may promote Stx2 associated disease by affecting Stx2 intestinal toxicity, uptake, and/or translocation into the systemic circulation resulting in intoxication of distal target organs. These studies may open the door to the development of novel therapeutic approaches for treating EHEC associated disease.

## Data Availability Statement

The datasets generated for this study are available on request to the corresponding author.

## Author Contributions

All authors listed have made a substantial, direct and intellectual contribution to the work, and approved it for publication.

### Conflict of Interest

The authors declare that the research was conducted in the absence of any commercial or financial relationships that could be construed as a potential conflict of interest.
