# Supplementary figures and images for: Endocytosis, Cytotoxicity, and Translocation of Shiga Toxin-2 Are Stimulated by Infection of Human Intestinal (HCT-8) Monolayers With an Hypervirulent E. coli O157:H7 Lacking stx2 Gene
Source: Front Cell Infect Microbiol. 2019 Nov 21;9:396. doi: 10.3389/fcimb.2019.00396 (PMC6881261; doi:10.3389/fcimb.2019.00396)

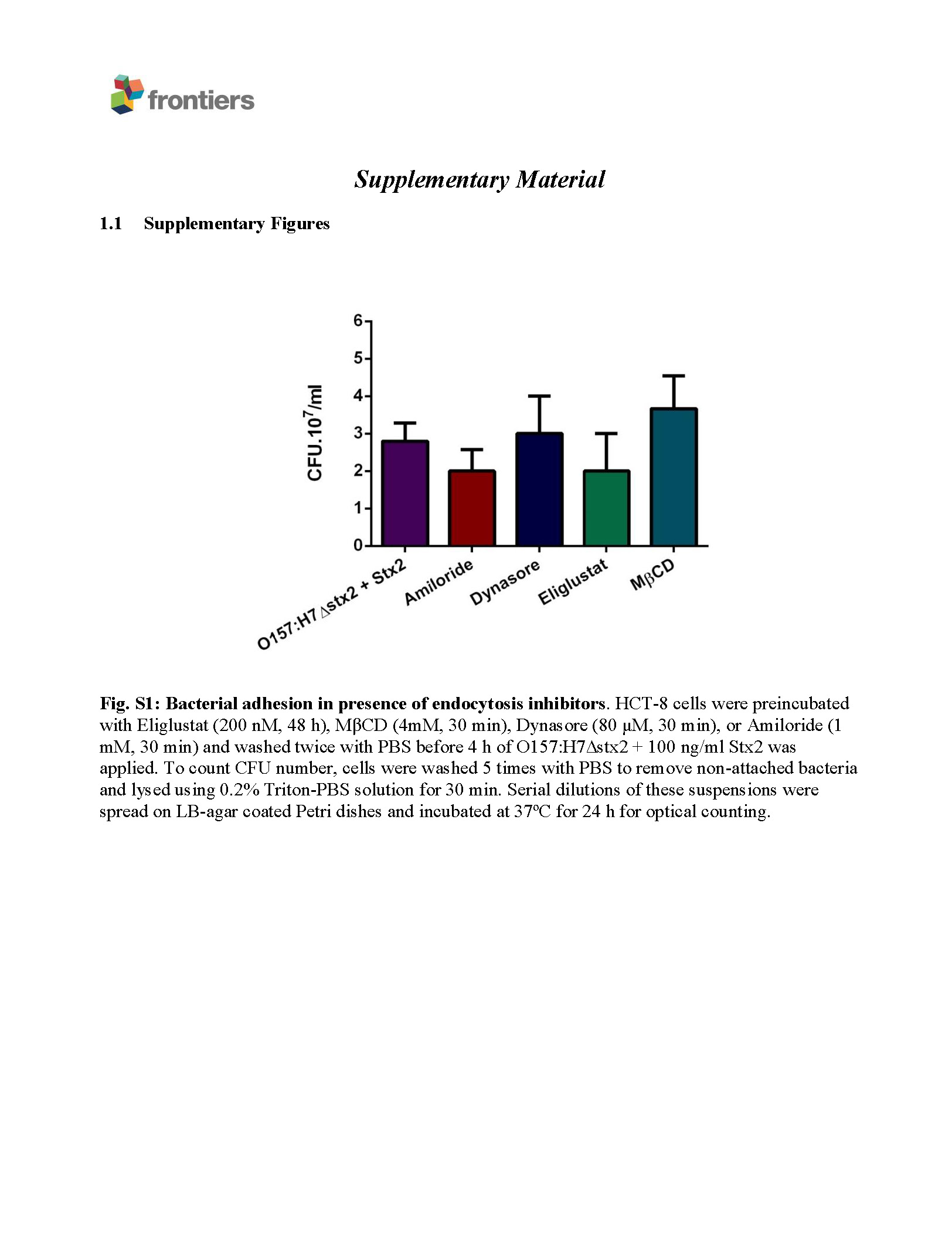

Supplement: Supplementary file 1 [file Image_1.JPEG]
